# A large dataset of detection and submeter-accurate 3-D trajectories of juvenile Chinook salmon

**DOI:** 10.1038/s41597-021-00992-x

**Published:** 2021-08-06

**Authors:** Jayson Martinez, Tao Fu, Xinya Li, Hongfei Hou, Jingxian Wang, M. Brad Eppard, Zhiqun Daniel Deng

**Affiliations:** 1 Pacific Northwest National Laboratory, Richland, Washington 99354 USA; 2 United States Army Corps of Engineers – Portland District, Portland, Oregon 97204 USA; 3 Department of Mechanical Engineering, Virginia Tech, Blacksburg, Virginia 24061 USA

**Keywords:** Environmental impact, Animal migration

## Abstract

Acoustic telemetry has been used extensively to study the behavior of aquatic animals. The Juvenile Salmon Acoustic Telemetry System (JSATS) is one such system; it was developed for studying juvenile salmonids but has been used to study numerous species. A recent innovation of the JSATS system is an acoustic transmitter that is small enough to be implanted through injection or small incision that doesn’t require sutures. Use of the JSATS system involves deploying cabled acoustic receivers at hydroelectric dams, or other structures, and autonomous acoustic receivers in free-flowing sections of a river. The raw detections from acoustic-tagged fish are processed to remove potential false positives. The clean detections (5,147,996 total) are used to generate detection events and to compute 3-D trajectories (403,900 total), which are used to assign fish to a passage route through a dam. Controlled field testing involving a high-accuracy Global Positioning System receiver is done to validate the submeter accuracy of the trajectories. The JSATS dataset could be reused for expanding the understanding of near-dam fish behavior.

## Abstract (summary)

**Table:** 

Measurement(s)	voluntary movement behavior • 3-D trajectory
Technology Type(s)	acoustic telemetry • Computation
Factor Type(s)	river • array type
Sample Characteristic - Organism	Oncorhynchus tshawytscha
Sample Characteristic - Environment	dam • freshwater biome
Sample Characteristic - Location	Little Goose Lock and Dam • Snake River • Columbia River • Lower Monumental Lock and Dam • McNary Dam • Bonneville Dam • Ice Harbor Lock and Dam • John Day Dam • Tucannon River

Machine-accessible metadata file describing the reported data: 10.6084/m9.figshare.14939169

## Background & Summary

Telemetry is often used to understand the effects of hydropower structures on fish species of concern. Acoustic telemetry has been used extensively to study fish and other aquatic animals, given the advantages of long detection ranges and the ability to accurately localize the tagged animals. One example of an acoustic telemetry system is the Juvenile Salmon Acoustic Telemetry System (JSATS; www.pnnl.gov/technology/jsats). JSATS was initially developed for the U.S. Army Corps of Engineers (USACE) to evaluate the behavior and survival of juvenile salmonids migrating past dams, through reservoirs, and along the lower Columbia River estuary to ocean entry^1–3^. It has since been used to study numerous fish species worldwide^4,5^.

**Fig. 1:**
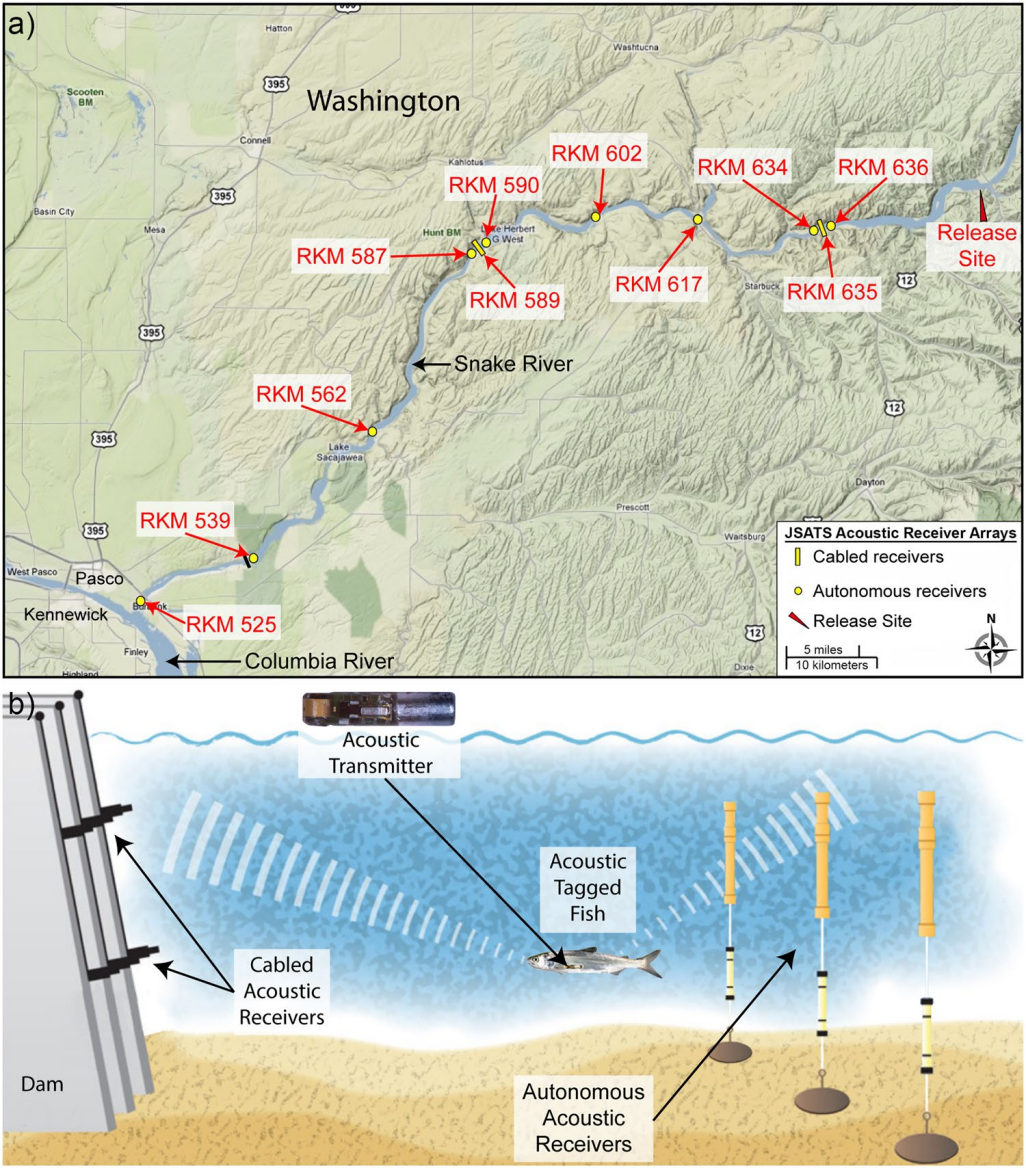
Field study design: (**a**) map showing the deployment of JSATS cabled and autonomous receiver arrays in the Snake River (Washington, USA; see Table 1 for array name); (**b**) schematic showing the key JSATS components of cabled acoustic receivers, autonomous acoustic receivers, and acoustic transmitters.

**Fig. 2:**
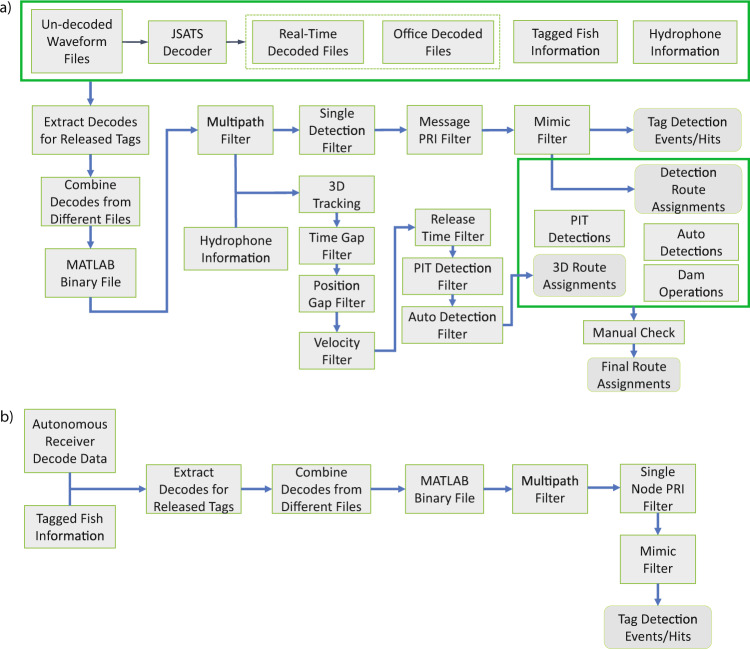
JSATS acoustic data processing for (**a**) cabled acoustic receiver data; (**b**) autonomous acoustic receiver data.

JSATS consists of acoustic microtransmitters^6–9^ (Fig. 1b), autonomous receivers^1,10^ (Fig. 1b), cabled receivers^2,3^ (Fig. 1b), data filtering algorithms^11^ (Fig. 2a,b), and advanced 3-D localization solvers^12^ (Fig. 2a). The type of acoustic receivers used in studies depends on the deployment location and the study needs. For applications where 3-D trajectories of tagged fish are needed to obtain detailed behavioral information, cabled hydrophone receiver arrays are typically used. These systems require extensive cabling to a centralized location that provides a 110 V AC power source and suitable environmental controls. For applications where 3-D trajectories are not required, or where suitable infrastructure to deploy cabled receivers doesn’t exist, autonomous receivers are used to detect the presence of tagged fish. Autonomous receivers are typically deployed immediately upstream and downstream of dams, as well as at locations between dams, for estimating survival and behavior.

Considerable effort has been expended to understand the biological effects of implanting acoustic transmitters in yearling Chinook salmon (*Oncorhynchus tshawytscha*), juvenile steelhead (*Oncorhynchus mykiss*), and subyearling Chinook salmon. The size and weight of JSATS microtransmitters used prior to this study met transmitter burden guidelines^13^ for most juvenile salmonids. However, a smaller transmitter would further reduce the possibility of adverse effects of implantation compared to previously available transmitters and would likely allow the scientific community to include smaller members of the population, therefor making the results obtained more representative of the population. With funding from the USACE, Pacific Northwest National Laboratory (PNNL) developed a revolutionary downsized transmitter^14–16^ for juvenile salmonids that meets the weight and volume targets for implantation by injection^17,18^, resulting in the first acoustic transmitter that can be implanted via injection instead of surgery^6^.

**Fig. 3:**
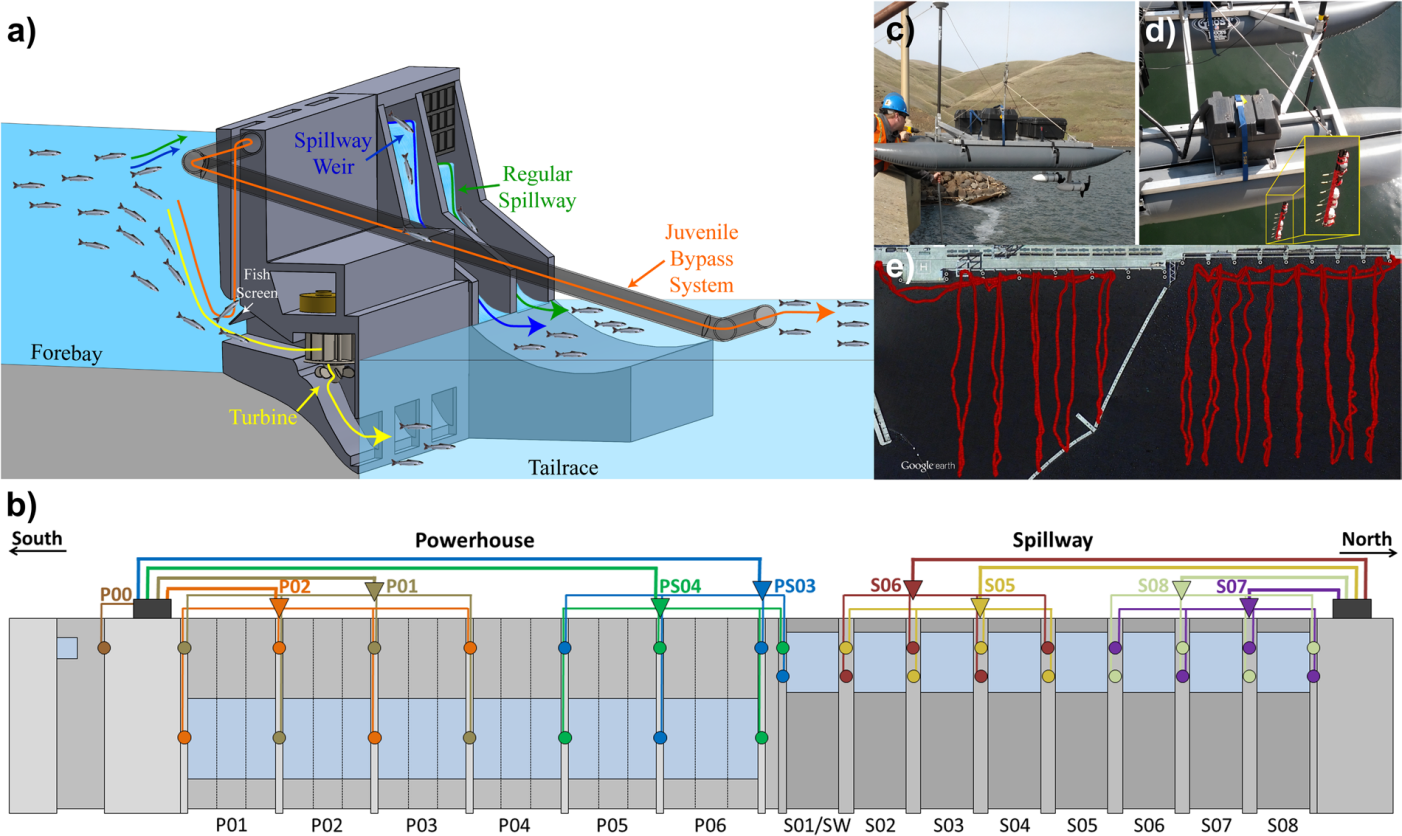
JSATS Cabled Hydrophone Array: (**a**) Schematic depicting dam subroutes; (**b**) elevation view schematic of the JSATS cabled acoustic array deployment at LGS Dam; (**c**) unmanned surface vessel with survey-grade GPS system used for the controlled field testing; (**d**) JSATS transmitters deployed from a 3 m-long steel pipe directly below the GPS antenna; (**e**) GPS trajectory of the controlled field testing at LGS Dam.

To evaluate the downsized JSATS injectable transmitter, a field study^11^ was conducted in which 682 subyearling Chinook salmon were tagged with the injectable acoustic transmitter and released upstream of Little Goose Dam (LGS) on the Snake River in Washington State, USA (Fig. 1a). JSATS cabled acoustic receiver arrays were deployed at LGS (Fig. 3b) and Lower Monumental Dam (LMN) to allow for 3-D tracking that facilitates assigning the passage route through the dam for each of the tagged fish. In addition to the cabled receiver arrays, several JSATS autonomous receiver arrays were deployed throughout the Snake (Fig. 1a) and Columbia rivers (Table 1) to estimate the cumulative mortality of the juvenile Chinook salmon as they migrate toward the Pacific Ocean. Each fish was also tagged with a passive integrated transponder (PIT) tag, for use with the PIT tag detectors deployed at many of the dams that the fish traverse on their way to the ocean.

**Table 1:** Locations of JSATS acoustic receiver arrays deployed in the Columbia and Snake Rivers in Oregon and Washington, USA.

Array Name	River Kilometer (RKM)	River	Array Type
LGS Forebay	636	Snake	Autonomous
LGS Dam	635	Snake	Cabled, PIT
LGS Tailrace	634	Snake	Autonomous
Lyons Ferry^a^	617	Snake	Autonomous
Ayer Boat Basin	602	Snake	Autonomous
LMN Forebay	590	Snake	Autonomous
LMN Dam	589	Snake	Cabled, PIT
LMN Tailrace	587	Snake	Autonomous
Snake River Road Launch	562	Snake	Autonomous
Ice Harbor Forebay	539	Snake	Autonomous
Ice Harbor Dam	538	Snake	PIT
Burbank	525	Snake	Autonomous
McNary Dam	470	Columbia	PIT
John Day Dam	347	Columbia	PIT
Bonneville Forebay	236	Columbia	Autonomous
Bonneville Dam	234	Columbia	PIT
Knapp Point (Knapp, WA)	152	Columbia	Autonomous

The locations of PIT detection arrays associated with juvenile bypass facilities are also provided. River kilometers are given relative to the mouth of the Columbia River.

^a^Li *et al*.^30^.

After collecting and decoding the raw transmitter detections from both cabled receiver arrays and each of the autonomous receiver arrays, advanced filtering algorithms (Fig. 2) were used to remove potentially false positive detections. False positives occur when an acoustic signal with sufficient energy to potentially be a tag transmission is collected, decoded to recover the unique ID, and initially assessed to determine if it is a valid JSATS acoustic transmission. To determine if a decoded signal could be from an acoustic tag, a parameter known as the cyclic redundancy check (CRC) is used. A JSATS tag transmission utilizes the final 8 bits of the signal to represent the CRC. Upon receiving and decoding the acoustic transmissions, the CRC can be computed from the decoded data and compared to the CRC included in the data transmitted. If the computed CRC values match it indicates that the decoded data is a valid JSATS tag transmission, however because only 8 bits are used a randomly computed CRC will be valid once out of 256 times. If an acoustic signal resulting from noise, or an incorrectly decoded transmission, has a valid CRC it is considered a false positive. If the false positives were included in the dataset it could result in an inability to solve for the location of the tag (if occurring alongside valid detections) or impossible migration histories. The cleaned set of transmitter decodes was used to define detection events that represent a period of nearly continuous detections for each transmitter at each array. For each of the cabled receiver arrays, the cleaned set of transmitter decodes was also used to 3-D localize tagged fish to assign passage routes through the dams. Controlled field testing, involving the use of a high accuracy Global Positioning System (GPS) receiver, was used to validate the accuracy of the 3-D trajectories. This dataset represents a relatively large sample set of subyearling Chinook salmon that were tagged with the downsized acoustic transmitter and subsequently released and detected at multiple locations on their journey to the Pacific Ocean. The dataset presented in this manuscript could be used in the future to deepen understanding of near-dam behavior and migration timing of subyearling Chinook salmon.

## Methods

### JSATS acoustic receiver deployments

Two types of acoustic receivers were deployed for this study: cabled and autonomous acoustic receivers (Table 1). These systems were deployed in the Snake River in eastern Washington State and in the lower Columbia River along the Oregon/Washington border. Two hydropower dams were outfitted with cabled acoustic receiver arrays: LGS and LMN. This allowed passage routes to be defined at each of these dams for each of the tagged fish. Transects of autonomous acoustic receivers (i.e., receiver arrays) were deployed immediately upstream and downstream of each of these dams for computing dam passage survival, and at several other locations to estimate cumulative mortality. From the release site to the final autonomous acoustic receiver array, fish detected at the final array will have passed through LGS, LMN, Ice Harbor, McNary, John Day, The Dalles, and Bonneville Dams. Each of these dams feature powerhouses with large, vertical Kaplan runners^19^ and spillways utilizing radial or vertical lift spillway gates. Most also include juvenile bypass systems (JBS; Table 1) and surface spill weirs^20^ to provide safer passage routes for fish.

Before deployment, all hydrophones and receivers were evaluated in an acoustic tank lined with anechoic materials at the PNNL Bio-Acoustics & Flow Laboratory (BFL^21^). The BFL is accredited by the American Association for Laboratory Accreditation to ISO/IEC 17025:2005, which is the international standard for calibration and testing laboratories. The accreditation scope (Certificate Number 3267.01) includes hydrophone sensitivity measurements and power-level measurements of sound sources for frequencies from 50 to 500 kHz for both military equipment and commercial components. The evaluation involved simulating transmissions from tags located at increasing distances. This allowed the performance of each receiver to be validated prior to deployment to ensure the expected detection range will likely be achieved.

#### Cabled receivers – deployment, hardware, and data processing

A JSATS cabled acoustic receiver system^2^ consists of up to four narrowband hydrophones; various types of hydrophone cables (e.g., four-channel “deck” cables, “y-blocks” that split the “deck” cables to individual connectors, and “wet” cables that run from the surface down into the water); a signal-conditioning, variable-gain amplifier; a data acquisition card that features a high-speed, analog-to-digital converter, a digital signal processor and field-programmable gate array; a GPS receiver for synchronizing time among multiple systems; a data acquisition computer; and software for detecting^22^ and decoding^23,24^ the acoustic waveforms. Deploying these systems within the forebay of a hydropower dam typically involves rigidly mounting slotted pipes to the upstream edge of the pier nose between the powerhouse and spillway bays. The cabled hydrophones are mounted on “trolleys” that have an L-shaped arm that protrudes and rides in the slot in the pipe and allows the hydrophone to be mounted pointing upstream. Conical baffles containing anechoic material are installed around the hydrophones to block noise coming from behind the hydrophone—either noise from the dam or reflections off the concrete. To obtain the locations of hydrophones that have been lowered below the water surface, survey equipment is used to sight the tip of the hydrophone as it is lowered down the pier nose. This provides the true direction and slope of the pier nose, which is used, along with the length of braided steel cable attached to the trolley to lower it into place, to calculate the 3-D location in space for each hydrophone. The individual “wet” cables for each hydrophone are routed to the forebay deck, where they are combined into “deck” cables carrying four signals using the “y-block” cables. The deck cables are routed to mobile trailers that house the acoustic receiver equipment. Acoustic beacons that send out JSATS tag-code signals every 15 to 60 s are deployed alongside several of the hydrophones across the array. These beacons are used primarily for quality control, to monitor (typically through the internet from an off-site location) the performance of each hydrophone to determine whether there is a reduction in performance so that any malfunctioning hydrophones can be repaired as soon as possible.

Two main programs run on the JSATS cabled receiver data acquisition computer. The first program is an energy-based detector software^22^ that collects the raw acoustic waveforms whenever the hydrophone signal meets a prescribed set of criteria. The second is decoder software^24^ that processes the waveforms saved by the detector to determine whether there is a valid detection (i.e., a decoded signal that has a valid CRC). If there is a valid detection, the decoded tag-code is saved to a text file, along with the detection time and other metadata. The detector software writes the binary waveform files to the hard drive using the *.bwm file type. The decoder is configured to wait for *.bwm files to be generated. Once a new *.bwm file is detected, the decoder will open the file, decode the data contained in the file, and then change the file extension to *.com to indicate that this file was decoded. If the detector saves data faster than the decoder can process it, for example at a hydraulic structure that is generating large amounts of acoustic noise (e.g., spillways with vertical lift gates), the decoder is configured to skip waveform files to avoid falling behind. Although these files may be skipped, the data contained within these files will still be used because the two different file extensions allow for readily identifying which files were not decoded in real time so that they can be decoded offline in a separate processing step after retrieving the data. The data is physically collected every 1–2 weeks, by swapping out the data collection hard drives. To make sure that all detection waveforms are processed by the decoder, the collected hard drives are put into data processing machines to decode any files that still have the *.bwm file extension. After confirming that all files have been decoded, either in real time or through post-processing after data retrieval, the decoded data from every hydrophone is checked for gaps in data. If a hydrophone is functional, there should be no large gaps in decoded data, since there are multiple stationary acoustic beacons deployed with the cabled hydrophone receiver array.

Data is filtered to remove potential false positive decodes. Data filtering for the JSATS cabled acoustic receivers (Fig. 2a) begins with a multipath filter, which removes decodes from multipath signal propagations (e.g., acoustic reflections off the surface/bottom). The multipath filter is used on the data from each individual hydrophone. Any decodes of the same tag-code that occur a very short time (e.g., typically <≈0.3 s) after an initial decode are removed, where the initial decode is assumed to be the original direct-path signal propagation. After the suspected multipath signals are removed, the remaining data from each hydrophone is combined into a single dataset and the decodes from each tag-code are grouped into “messages” suspected to be from the same tag transmission based on the detection time. A single detection filter removes any messages that were only detected by a single hydrophone, since the hydrophone spacing should enable any real signal from a transmitter within the detection range to be detected by multiple hydrophones. A message ping-rate interval (PRI) filter is then used on the data to remove messages that do not follow the expected transmission pattern inherent to all JSATS transmitters. The message PRI filter also groups messages into “events” of nearly continuous decodes of a tag-code. A predictable corruption of the tag-code bit pattern (e.g., a bit inversion at a specific bit position) can cause the incorrect decoding of a real tag-code signal and the generation of a different valid tag-code (i.e., a valid CRC). The final filter used on the dataset is a mimic filter, which removes these “mimic” decodes which result from the method to encode the data to be transmitted and is thus inherent to all JSATS acoustic receivers. To obtain the event history of each tag-code, the dataset is checked for events from known mimic tag-codes that occur during the same period. If an event is suspected to be from a mimic tag-code, the 3-D trajectories for the two tag-codes are compared to determine whether they appear to originate from the same spatial location. The result of this data filtering is a clean list of event histories for each tag-code, which is used in subsequent analysis of detection histories. A more detailed description of the data filtering process, including assessments of field detection probability and false-positive detection probability, are provided in Deng *et al*.^11^.

Computing the 3-D trajectories starts with the data from the multipath filter. The accurately time-stamped, decoded data is used to calculate the time difference of arrival (TDOA) between the detection times of a transmission on each of the different hydrophones. The TDOAs are used with the 3-D location of each hydrophone and water temperature data to compute the sound speed, to calculate the source location using an approximate maximum likelihood solver^12^. At each dam, the geographic coordinates of the hydrophones are converted to a local Cartesian coordinate system, in which the *X*-axis extends out into the forebay, the *Y*-axis runs parallel to the dam, and the *Z*-axis is normal to the water surface. Once the raw 3-D trajectories have been computed for each tag-code, a series of filters removes 3-D tracked points that are outliers. These filters include ones to remove points that are too far away spatially from adjacent points (>45 m), too far away temporally from adjacent points (>10 min), and that result in unrealistic velocities (~2 m/s for the size of fish we studied). Further quality assurance filters remove points that occur before a transmitter was released, after the PIT tag in the fish is detected downstream, and after the acoustic transmitter is detected by downstream autonomous receiver arrays.

Once the 3-D trajectories have been computed, they are used to assign passage routes through the dam for each tagged fish (Fig. 3a). The route assignment for each tagged fish is divided into three parts: main route, subroute, and hole (Table 2). The main route describes the part of the general dam structure through which the tagged fish passed. This includes the powerhouse, the spillway, and a generic category, “dam,” which is used for rare scenarios where there is confidence that the fish was physically present but a lack of confidence in specifically where the fish passed the dam. The subroute further divides the main passage route into different subcategories. For a main route of “spillway,” the two subroutes are the traditional (deep) spillbays and special surface weirs (surface spillbays^20^). The surface weirs reside within one of the spillbays and assignment to either of these two subroutes is made directly using the acoustic telemetry results. For a main route of “powerhouse,” the two subroutes are turbine and JBS. Assignment of the JBS subroute requires that the PIT tag of a fish assigned to the powerhouse was detected by the PIT tag readers within the JBS system; otherwise the fish is assigned the turbine subroute. PIT tag detections at dams where cabled hydrophone arrays have been deployed can be used to assign the JBS subroute, and PIT tag detections at the other dams along the migration route can serve as additional detection events. The hole assignment defines the specific powerhouse intake or spillbay where the passage occurred.

**Table 2:** Dam passage routes (main route, subroute, and hole) at LGS and LMN Dams.

Dam	Main Routes	Subroutes	Hole (direction of numbering; T = turbine; B = Spillbay)
LGS	powerhouse	turbine	T01-T06 (south-north)
LGS	powerhouse	JBS	T01-T06 (south-north)
LGS	spillway	regular_spillway	B01-B07 (south-north)
LGS	spillway	spillway_weir	B01
LGS	dam	N/A	N/A
LMN	powerhouse	turbine	T01-T06 (north-south)
LMN	powerhouse	JBS	T01-T06 (north-south)
LMN	spillway	regular_spillway	B01-B07 (south-north)
LMN	spillway	spillway_weir	B08
LMN	dam	N/A	N/A

Passage routes are assigned using the last 3D tracked location and the last detection. Two methods are used because the ability to consistently track the transmitter can diminish as the tagged fish approaches or passes through the plane containing the hydrophones, and the last decoded transmission could be later than the last 3-D tracked location.

When the last 3-D tracked location is used, a route is assigned according to whether the last tracked point is within a specific area. This area spans the entire dam plus 25 m on each side and extends from the dam face to 30 m upstream into the forebay. If the last tracked point is within this boundary, the 3-D track passage route is assigned to the bay corresponding to the *Y* coordinate in the local dam coordinate system. If the last tracked point is outside the piers on either side of the dam, the passage route is assigned to the nearest bay.

Route assignment based on the last detection uses the last transmission that was detected by multiple hydrophones. The detections associated with this transmission are sorted by time, and the pier numbers for the two hydrophones on different piers that first detected this transmission are averaged; the passage bay corresponding to this average pier is assigned as the last detection passage route. The default final route assignment is the 3-D tracked route assignment. However, when the two methods indicate different main routes, subroutes, or a different hole that is more than two bays away, the 3-D tracks are manually reviewed, and a decision is made regarding which method should be relied on for the final route assignment.

After the final route assignment, a final quality assurance step is to compare the final route assignment to the dam operations. In case a tag-code has been assigned to a closed passage route, the 3-D tracks are reviewed to consider the trajectory of the tagged fish and the location of the nearest open passage route. As previously mentioned, the ability to consistently track a transmitter is diminished as it approaches or passes through the plane containing the hydrophones. An example of when a tagged fish could initially be assigned to a closed passage route would be when a passage route with a strong attractive flow (e.g., surface spill weir) is adjacent to a closed passage bay.

#### Autonomous receivers – deployment, hardware, and data processing

A JSATS autonomous receiver (SR5000, Advanced Telemetry Systems [ATS], USA), along with the necessary deployment accessories, consists of a hydrophone that is connected to a cylindrical, positively buoyant, self-contained, battery-powered, autonomous acoustic receiver; a submerged buoy line; an acoustic release; a braided stainless-steel cable; and a steel anchor. These receivers are typically deployed according to the methods described by Titzler *et al*.^10^, which involves using a 34 kg or 57 kg (depending on flow) steel anchor to deploy the autonomous receiver system to the river bottom. The anchor is attached to the release side of an acoustic release using a braided stainless-steel cable. The fixed end of the acoustic release is attached to the autonomous receiver using a submerged buoy line. When the acoustic release is remotely triggered, it detaches from the anchor line, and the combined buoyancy of the acoustic receiver and the submerged buoy line bring the system up to the surface. To maintain the receiver orientation in the water column during deployment, a thin plastic sheet is folded around the cylindrical body of the receiver, creating an airfoil-like shape that keeps the receiver oriented in the flow direction. Attached to each autonomous receiver is a JSATS beacon that is similar to the JSATS beacons deployed with the cabled hydrophone receiver arrays and used in the same way. The length of time that the JSATS autonomous receivers can be deployed is largely dependent on the battery life, with data retrieval and battery changes typically done every 2–3 weeks.

Although it is possible to use autonomous receivers to conduct 3-D tracking, the process is much more challenging than using the cabled hydrophone receivers because the receivers are not fixed at well-defined locations (e.g., changes in river currents could change the receiver’s depth and horizontal location relative to the anchor) and are not time synchronized with each other. The autonomous receivers are typically used to detect presence of tagged fish, although recent research has investigated methods to improve the ability to conduct 3-D tracking^25^. Deploying an autonomous receiver array entails deploying several receivers in a line spanning the width of a river with the detection ranges of the receivers overlapping slightly. This creates a virtual detection gate that can be used to determine when a tagged fish passes through this location in the river. In addition to simply determining the migration timing of tagged fish, the autonomous receiver arrays are typically used for analyzing dam passage survival and near-dam behavior (e.g., forebay residence time, tailrace egress time).

The data collected by the autonomous receivers is filtered similarly to data from the cabled receivers (Fig. 2b). The primary difference is that the data from each individual autonomous receiver is processed entirely by itself. As a result, the single-hydrophone filter is not used, and a single-node PRI filter is used instead of the message PRI filter.

After the event histories for both the cabled and the autonomous acoustic receivers have been determined, individual routes were cross-checked by tracing the chronology of detections of every tagged fish as it was detected along the river in the sequence of acoustic receiver arrays. Upstream movement past a dam or out-of-sequence detections were deemed anomalous detection events. These anomalous detection events could be a few receptions resulting from noise or repeated detections of a transmitter that had been dropped near a receiver array after fish or bird predation. If the apparent behavior was impossible for a live fish, the anomalous detection was excluded from the detection history used for subsequent analysis.

### JSATS transmitters

The injectable transmitters used in this study (Fig. 1b) were manufactured by PNNL. Each transmitter (model microV2^6^, which is licensed to, and currently commercially available from, Advanced Telemetry Systems as Model SS400) was 15 mm long, had an outside diameter of 3.35 mm, a volume of 0.111 mL, and a mass of 0.216 g in air and 0.105 g in water. The transmitters are generally cylindrical; excess epoxy was eliminated to reduce the weight, and epoxy surrounding the transducer element was minimized. The transmitters had a nominal transmission rate of 1 pulse every 4.2 s. Nominal transmitter life was expected to be about 28 d at a 4.2 s pulse rate. The acoustic signal is transmitted using a carrier frequency of 416.7 kHz, a source level of approximately 156 dB (ref. to 1 µPa at 1 m), and a total signal duration of 477 µs. The transmitter emits a uniquely coded 31-bit signal^2^, resulting in more than 65,000 individual tag-codes, using binary phase-shift keyed (BPSK) signal encoding.

Each fish also bore a PIT tag (HPT12, Biomark, USA; 12.5 mm x Ø2.03 mm; 0.106 g in air). PIT tag detections were used to assign fish to passage through the JBS at LGS and LMN to distinguish between fish that were assigned to a main route of powerhouse and the turbine or JBS subroutes.

### Tagged fish

For this study, 682 subyearling Chinook salmon (*Oncorhynchus tshawytscha*) were tagged with the injectable acoustic transmitter and released upstream of LGS Dam on the Snake River in Washington State, USA (Fig. 1a). The fish were obtained from the JBS at LMN Dam and selected using existing fish screening criteria utilized in previous juvenile salmon survival studies^26^. The fish selected for the study were held in holding tanks for 18 to 30 hours prior to tagging, and for 10 to 25 hours after tagging prior to release. The size criteria for tagged fish was also identical to other recent juvenile salmon survival studies^26^. For this study the fork-lengths ranged from 95 to 143 mm, and the weights ranged from 7.5 to 29.3 g (see Tagged Fish Data for information on each tagged fish).

### Tagging procedure

While each anesthetized fish was at the data station for recording physical parameters, a second person inserted both a disinfected PIT tag and an injectable acoustic transmitter, assigned to a specific fish, into a sterilized 8-gauge stainless-steel hypodermic needle^17^. First, the injectable transmitter was placed into the needle, battery-end first. The PIT tag was then also inserted in the same needle. A sanitized plastic cap was then placed over each end of the needle to retain the tags. Once both tags had been placed in the needle, the tag loaded needle was handed to the surgeon working at the tagging station. Additional details for the tagging procedure are documented by Deng *et al*.^11^.

### Release procedure

The fish implanted with the injectable acoustic transmitters were released using the same methods as fish tagged with commercially available acoustic transmitters for a separate large-scale survival study^26^. All fish were tagged at LMN and transported in insulated totes by truck to the single release site (Fig. 1a). There were five release locations across the river at the release site, and equal numbers of fish were released at each of the five locations. Releases occurred for 11 consecutive days (between 22 June and 2 July, 2013) and were staggered between day and night.

### Data management

Use of JSATS can generate a large volume of data. An integrated suite of science-based tools known as the Hydropower Biological Evaluation Toolset (HBET; https://hydropassage.org/hbet)^27^ was developed to assist the characterization of hydraulic conditions at hydropower structures and to understand the potential impacts on aquatic life. HBET was initially developed to be utilized to facilitate use of the autonomous sensor technology known as Sensor Fish^28^. HBET allows researchers to use previously collected Sensor Fish data to design studies to evaluate hypotheses, archive field-collected data, process raw sensor data, compare different hydraulic structures or operating conditions, and to estimate the biological response for species with known dose-response relationships. More recently, HBET was adapted to also provide the functionality of archiving new or previously collected acoustic telemetry data and to produce visualizations from that data. Although it is not necessary to visualize the data set associated with this manuscript, PNNL offers free government and academic use of the HBET software package in the U.S. and a free 90-day trial version of the package to interested parties.

## Data Records

The data records for this study^29^ are a series of text files covering the study design and the subsequent results. The description of the data records is organized into categories.

### Autonomous receiver deployment data

Filename: *Autonomous_Receiver_Deployment_Data.csv*

Format: Comma-separated-value text file

Data Columns:

(A) Node_ID: Unique identification of an autonomous receiver deployment that contains the serial number and the deployment date
(B) Node_SN: Serial number of the autonomous receiver
(C) Latitude_(NAD83): Latitude of the autonomous receiver in the NAD83 geographic coordinate system
(D) Longitude_(NAD83): Longitude of the autonomous receiver in the NAD83 geographic coordinate system
(E) River Kilometer: Distance along the Columbia River from the Pacific Ocean to the location of the autonomous receiver
(F) Depth_(m): Depth below the water surface to the autonomous receiver’s hydrophone
(G) Deploy_Date: Date and time when the autonomous receiver was deployed
(H) Recovery_Date: Date and time when the autonomous receiver was recovered
(I) Location_ID: Location identification of the autonomous receiver deployment, where “CR” or “SR” stands for Columbia River or Snake River, the next four digits represent the river kilometer from the Pacific Ocean (SR) or where the Snake River joins the Columbia River (CR), and the two digits after the underscore represent the position across the breadth of the river.
(J) Start_Sample: Data and time when the autonomous receiver starts sampling
(K) End_Sample: Data and time when the autonomous receiver ends sampling
(L) Comments: Additional information related to the autonomous receiver deployment

### Cabled hydrophone receiver deployment data

Filenames: *LGS_Hydrophone_Configuration.csv* and *LMN_Hydrophone_Configuration.csv*

Format: Comma-separated-value text file

Data Columns:

(A) Sys_#: Index number of the cabled hydrophone receiver system
(B) Sys_Name: Name of the cabled hydrophone receiver system; contains a letter indicating at which structure the hydrophones are placed (P – powerhouse, S – spillway, PS – both powerhouse and spillway) and the index number of the cabled hydrophone receiver system
(C) Location: Location of the hydrophone; usually contains a letter indicating at which structure the hydrophone is deployed (P – powerhouse, S – spillway), one bay number or two bay numbers separated by an underscore, indicating between which two bays of the dam the hydrophone is located, and a letter indicating the relative depth where the hydrophone is placed (D – deep, S – shallow). FLS and FLN represent fish ladder south and fish ladder north, respectively. RSW represents hydrophone deployed on structures in front of the removable spill weir.
(D) Node_Position: Relative depth where the hydrophone is deployed (D – deep, S – shallow)
(E) Channel: Index number of the hydrophone channel of the cabled hydrophone receiver system
(F) Phone_ID: Index number of the hydrophone
(G) Pier_ID: Index number of the dam pier where the hydrophone is deployed
(H) Phone_Name: Name of the hydrophone; usually contains a letter indicating at which structure the hydrophone is deployed (P – powerhouse, S – spillway), one bay number or two bay numbers separated by an underscore, indicating between which two dam bays the hydrophone is located, and a letter indicating the depth where the hydrophone is placed (D – deep, S – shallow). FLS and FLN represent fish ladder south and fish ladder north, respectively. RSW represents hydrophone deployed on structures in front of the removable spill weir.
(I) Latitude_(NAD83): Latitude of the hydrophone in the NAD83 geographic coordinate system
(J) Longitude_(NAD83): Longitude of the hydrophone in the NAD83 geographic coordinate system
(K) Easting_(m; NAD83 – WA South): Easting coordinate (m) of the hydrophone in the NAD83 Washington South state plane coordinate system
(L) Northing_(m; NAD83 – WA South): Northing coordinate (m) of the hydrophone in the NAD83 Washington South state plane coordinate system
(M) Elevation_(m AMSL): Elevation (m) of the hydrophone above mean sea level
(N) X_(m): Dam coordinate (m) of the hydrophone in the direction normal to the dam face
(O) Y_(m): Dam coordinate (m) of the hydrophone in the direction parallel to the dam
(P) Z_(m): Dam coordinate (m) of the hydrophone in the direction normal to the water surface

### Tagged fish data

Filename: *Tagged_Fish_List.csv*

Format: Comma-separated-value text file

Data Columns:

(A) Tag_ID: Acoustic tag unique identification code
(B) PIT_ID: PIT tag unique identification code.
(C) Tag_Release_Date: Date and time when the tagged fish was released
(D) PRI: Ping rate interval of the tag, in seconds, at activation
(E) Fish_Tagging_Date: Date and time when the fish was tagged
(F) Fish_Length_at_Tagging_(mm): Length of the fish at tagging in millimeters
(G) Fish_Weight_at_Tagging_(g): Weight of the fish at tagging in grams

### PIT detection data

Filename: *PIT_Detection_Data.csv*

Format: Comma-separated-value text file

Data Columns:

(A) Tag_ID: Acoustic tag unique identification code
(B) PIT_ID: PIT tag unique identification code
(C) First_PIT_Datetime: Date and time when the PIT detections start
(D) Last_PIT_Datetime: Date and time when the PIT detections start
(E) PIT_Site: The location where the PIT tag detection occurred
(F) PIT_Site_Description: Description of the location where the PIT detection occurred
(G) PIT_Site_Basin: River basin where the PIT detection occurred
(H) PIT_Site_Subbasin: River subbasin where the PIT detection occurred
(I) PIT_Site_Type: Type of PIT detection site
(J) PIT_Site_Latitude_(NAD83): Latitude of the PIT detection site in the NAD83 geographic coordinate system
(K) PIT_Site_Longitude_(NAD83): Longitude of the PIT detection site in the NAD83 geographic coordinate system
(L) PIT_Site_RKM: Distance along the Columbia and Snake River from the Pacific Ocean to the location of the PIT detection site
(M) Fish_Characteristics_at_Tagging: Notes on the fish characteristics at the time of tagging
(N) PIT_Tagging_Date: The date that the PIT tagging occurred
(O) Fish_Length_at_Tagging_(mm): Length of the fish at tagging in millimeters
(P) Fish_Weight_at_Tagging_(g): Weight of the fish at tagging in grams

### Cabled receiver event data

Filenames: *LGS_Event_Data.csv* and *LMN_Event_Data.csv*

Format: Comma-separated-value text file

Data Columns:

(A) Phone_Name: Name of the hydrophone; usually contains a letter indicating at which structure the hydrophone is deployed (P – powerhouse, S – spillway), one bay number or two bay numbers separated by an underscore, indicating between which two dam bays the hydrophone is located, and a letter indicating the depth where the hydrophone is placed (D – deep, S – shallow). FLS and FLN represent fish ladder south and fish ladder north, respectively. RSW represents hydrophone deployed on structures in front of the removable spill weir. The hydrophone listed is the first hydrophone that detected the last message transmitted by the tag
(B) Tag_ID: Acoustic tag unique identification code
(C) Event_ID: Index number of the detection event for each tag
(D) First_Computed_Datetime: Date and time when the detection event starts
(E) Last_Computed_Datetime: Date and time when the detection event ends
(F) Number_Messages: Number of messages (i.e., tag transmissions) in the detection event

### Autonomous receiver event data

Filenames: *Autonomous_Receiver_Event_Data.csv*

Format: Comma-separated-value text file

Data Columns:

(A) Node_ID: Unique identification of an autonomous receiver deployment that contains the serial number and the deployment date
(B) Tag_ID: Acoustic tag unique identification code
(C) First_Computed_Datetime: Date and time when the detection event starts
(D) Last_Computed_Datetime: Date and time when the detection event ends
(E) Number_Messages: Number of messages (i.e., tag transmissions) in the detection event

### 3-D Trajectory data

Filenames: *LGS_3D_Tracks.csv* and *LMN_3D_Tracks.csv*

Format: Comma-separated-value text file

Data Columns:

(A) Tag_ID: Acoustic tag unique identification code
(B) Time: Date and time when the tag was detected
(C) Latitude_(NAD83): Latitude of the tag in the NAD83 geographic coordinate system
(D) Longitude_(NAD83): Longitude of the tag in the NAD83 geographic coordinate system
(E) Easting_(m; NAD83 – WA South): Easting coordinate (m) of the tag in the NAD83 Washington South state plane coordinate system
(F) Northing_(m; NAD83 – WA South): Northing coordinate (m) of the tag in the NAD83 Washington South state plane coordinate system
(G) Elevation_(m AMSL): Elevation of the tag above mean seal level
(H) X_(m): Dam coordinate (m) of the tag in the direction normal to the dam face
(I) Y_(m): Dam coordinate (m) of the tag in the direction parallel to the dam
(J) Z_(m): Dam coordinate (m) of the tag in the direction normal to the water surface

### Route assignment data

Filenames: *LGS_Passage_Routes.csv* and *LMN_Passage_Routes.csv*

Format: Comma-separated-value text file

Data Columns:

(A) Tag_ID: Acoustic tag unique identification code
(B) Route: Primary route of the tagged fish through the dam (e.g., spillway, powerhouse)
(C) Subroute: Subroute of the tagged fish through the dam (e.g., regular_spillway, spillway_weir, turbine, JBS)
(D) Passage_Hole: Specific passage bay by which the tagged fish passed through the dam; usually contains a letter indicating the passage structure (B – spillbay, T – turbine) and a number indicating from which bay the tag exits
(E) Array_Name: Name of the array; usually the three-letter abbreviation of the dam

## Technical Validation

### Controlled field testing

Technical validation for the 3-D tracking and the subsequent dam passage route assignments was done by conducting controlled field testing at LGS on 14 August 2013 using a 2.7 m-long unmanned surface vessel (USV; i.e., remotely operated boat; Fig. 3c). The USV was developed specifically for evaluating the accuracy of the 3-D tracking in the forebays of dams equipped with JSATS cabled arrays^2,3^. It is powered by two 55 lb-thrust electric trolling motors (EM 55, Minn Kota, Mankato, Minnesota, USA) powered by dual 12 V lead-acid, deep-cycle batteries. A 3 m-long steel pipe was mounted on the USV and used for positioning JSATS transmitters at a fixed depth below the USV. Attached to the pipe was an injectable transmitter programmed with a 3 s pulse interval. The transmitter was secured to the pole in a way that kept it facing toward the aft end of the USV (Fig. 3d). Before the controlled field testing, the transmitter was tested to obtain the full 360° directivity (beam pattern) about each axis. The locations of the transmitter were obtained through a real-time kinematic GPS system (Trimble GeoExplorer, Trimble Navigation Ltd., Sunnyvale, California, USA) and depth sensor (HOBO U20-001-03, Onset Computer Corporation, Bourne, Massachusetts, USA), which provided benchmark measurements for comparison with the 3-D-tracked locations.

In front of each turbine and spillway bay, the USV was driven slowly out to approximately 150 m from the upstream face of the dam and back, at an average speed of 0.28 m/s (Fig. 3e). The boat was driven so that the transmitters were always pointed toward the dam. The 3-D trajectories were calculated for each of the acoustic transmitters. To perform the analysis, the drifts toward and away from the dam were separated into 10 m bins. The detection efficiency, tracking efficiency, and tracking errors were computed for each of the bins. Detection efficiency was evaluated as the number of valid detections divided by the number of transmissions. Tracking efficiency was evaluated as the number of successful 3-D-tracked locations divided by the number of transmissions. The errors were assessed in terms of median (Eqs. 1–3) and root-mean-square (RMS; Eqs. 4–6) values of the differences between the GPS measurements and the source locations computed using specialized MATLAB (MATLAB 2013, MathWorks Inc., Natick, Massachusetts, USA) scripts similar to those described in Deng *et al*.^3^.

1 $$\Delta {x}_{i}=\left|{x}_{i}^{3D}-{x}_{i}^{GPS}\right|,\quad i=1,\ldots N$$

2 $$\Delta {y}_{i}=\left|{y}_{i}^{3D}-{y}_{i}^{GPS}\right|,\quad i=1,\ldots N$$

3 $$\Delta {z}_{i}=\left|{z}_{i}^{3D}-{z}_{i}^{GPS}\right|,\quad i=1,\ldots N$$

4 $${RMS}_{x}=\sqrt{\frac{1}{N}{\sum }_{i=1}^{N}\Delta {x}_{i}^{2}}$$

5 $$RM{S}_{y}=\sqrt{\frac{1}{N}{\sum }_{i=1}^{N}\Delta {x}_{y}^{2}}$$

6 $$RM{S}_{z}=\sqrt{\frac{1}{N}{\sum }_{i=1}^{N}\Delta {z}_{i}^{2}}$$

where *x*, *y*, and *z* are the cartesian coordinates in the coordinate system aligned with the dam; *N* is the number of points tracked, *3D* indicates the coordinate associated with the 3-D tracking from acoustic data; and *GPS* indicates the coordinates linearly interpolated from the GPS data at the moment of the acoustic tracked location.

### Detection and tracking efficiency

The detection efficiency of the injectable transmitter (Table 3) was greater than 97% out to 140 m from the dam. The tracking efficiency of the injectable transmitter (Table 3) was also high. Out to 110 m, the tracking efficiency was above 96%, and it decreased to approximately 50% at 140 m.

**Table 3:** Detection efficiency, tracking efficiency, median errors, and RMS errors for the controlled field testing conducted at LGS Dam.

Distance (m)	Detection Efficiency	Tracking Efficiency	Median *X*-Error (m)	Median *Y*-Error (m)	Median *Z*-Error (m)	RMS *X*-Error (m)	RMS *Y*-Error (m)	RMS *Z*-Error (m)
10	99.3%	99.3%	0.15	0.57	0.14	0.63	0.57	0.27
30	99.7%	99.7%	0.13	0.58	0.31	0.42	0.58	0.50
100	98.5%	98.3%	0.16	0.63	2.58	0.62	0.66	2.82
120	99.4%	93.4%	0.15	0.59	3.59	0.70	0.63	3.78
140	99.1%	50.4%	0.12	0.59	4.01	0.81	0.65	4.17

### Three-dimensional tracking error

Three-dimensional tracking errors were assessed in terms of the median and the RMS difference between the GPS measurements and the computed locations (Table 3). For the median errors, the *X*-axis (distance from dam) errors were found to be relatively constant at about 0.15 m out to 150 m away, the *Y*-axis (location along dam) errors were constant at about 0.6 m out to 150 m away, and the *Z*-axis (depth) errors varied from about 0.1 m at 10 m away to 5 m at 150 m away.

The RMS errors were similar to the median errors, with the *Z*-axis errors being the largest. The *X*-axis errors were about 0.5 m out to 80 m away and increased to 1–2 m at 150 m away, the *Y*-axis errors varied from 0.6 m at 10 m away to 0.7 m at 150 m away, and the *Z*-axis errors varied from 0.3 m at 10 m away to 5 m at 150 m away. The relatively large *Z*-axis errors arose from a large temperature variation with water depth in August and use of the average temperature for 3-D tracking. The *Z*-axis errors could be significantly reduced by using a more realistic temperature distribution and a more sophisticated tracking algorithm, which was not in the scope of this study.

## Data Availability

**MATLAB scripts**. To help facilitate the use of the dataset provided in this manuscript, custom MATLAB scripts are provided (MATLAB_scripts.zip) that read the data files, produces a set of files that can be manually reviewed, and serves as a starting point for interested parties to modify the scripts to further investigate the data set. These scripts are to be used by running them from within the same working directory as the data files. The provided scripts perform the following actions: • create_3D_track_plots.m ○ Reads data from the following files: ▪ Tagged_Fish_List.csv ▪ LGS_Hydrophone_Configuration.csv ▪ LMN_Hydrophone_Configuration.csv ▪ LGS_3D_Tracks.csv ▪ LMN_3D_Tracks.csv ○ If the MATLAB installation includes the Mapping Toolbox KML files will be generated that can be opened in Google Earth: ▪ Files to display the location of the hydrophones at LGS and LMN. ▪ A file for each tag detected at each dam that displays up to the last 1000 3D tracked locations. ○ For each tag detected at each dam a multi-panel plot will be generated that displays: ▪ Upper-left: XY coordinates of the cabled hydrophones and the 3D track for the tagged fish specified ▪ Upper-right: Time vs. X-location ▪ Lower-left: Time vs. Y-location ▪ Lower-right: Time vs. Z-location • create_event_history_plots.m ○ Reads data from the following files: ▪ Tagged_Fish_List.csv ▪ Autonomous_Receiver_Deployment_Data.csv ▪ Autonomous_Receiver_Event_Data.csv ▪ LGS_Event_Data.csv ▪ LMN_Event_Data.csv ○ For each tag that had an acoustic detection event, a plot was generated that shows the number of river kilometers from the Pacific Ocean against the days since the tagged fish was released. ▪ Since autonomous receivers are processed on an individual basis there are several overlapping events for each autonomous receiver array. **HBET JSATS C# Source code**. In addition to the simple, readily adaptable, MATLAB scripts provided for visualizing the data set and serving as a starting point for additional analysis, the C# source code for the JSATS features integrated into the HBET software are also provided. The source code was developed using C# via Microsoft Visual Studio 2019. To install and operate the C# code, the users must install Microsoft SQL Server express 2012 and attach the database file that contains the information contained within the data set (HBET_Database_Files.zip). The data handling codes are contained within a compressed folder included in the data repository (HBET_Source_Code.zip). The provided source code files perform the following functionality: • JSATSFishDectionForm.* ○ Used to display fish detection information • JSATSQueryForm.* ○ Used to retrieve data from the database • JSATSStartUpForm.* ○ Main form of the application • JSATSStudyManagement.* ○ Used to manage studies • JSATSUploadFileForm.* ○ Used to upload data files • TrackForm.* ○ Used to display fish migration information
